# Intramolecular hydrogen transfer in DNA induced by site-selective resonant core excitation†

**DOI:** 10.1039/d1cp05741c

**Published:** 2022-03-09

**Authors:** Xin Wang, Sivasudhan Rathnachalam, Vicente Zamudio-Bayer, Klaas Bijlsma, Wen Li, Ronnie Hoekstra, Markus Kubin, Martin Timm, Bernd von Issendorff, J. Tobias Lau, Shirin Faraji, Thomas Schlathölter

**Affiliations:** Zernike Institute for Advanced Materials, University of Groningen Groningen The Netherlands s.s.faraji@rug.nl t.a.schlatholter@rug.nl; Abteilung für Hochempfindliche Röntgenspektroskopie, Helmholtz-Zentrum Berlin für Materialien und Energie Berlin Germany; Physikalisches Institut, Universität Freiburg Freiburg Germany

## Abstract

We present experimental evidence for soft X-ray induced intramolecular hydrogen transfer in the protonated synthetic tri-oligonucleotide *d*(^F^UAG) in the gas-phase (^F^U: fluorouracil). The trinucleotide cations were stored in a cryogenic ion trap and exposed to monochromatic synchrotron radiation. Photoionization and photofragmentation product ion yields were recorded as a function of photon energy. Predominanly glycosidic bond cleavage leading to formation of nucleobase-related fragments is observed. In most cases, glycosidic bond cleavage is accompanied by single or double hydrogen transfer. The combination of absorption-site-sensitive soft X-ray spectroscopy with fragment specific mass spectrometry allows to directly relate X-ray absorption site and fragmentation site. We observe pronounced resonant features in the competition between single and double hydrogen transfer towards nucleobases. A direct comparison of experimental data with time-dependent density functional theory calculations, using short range corrected hybrid functionals, reveal that these hydrogen transfer processes are universal and not limited to population of particular excited states localized at the nucleobases. Instead, hydrogen transfer can occur upon X-ray absorption in any nucleobase and in the DNA backbone. Resonances seem to occur because of site-selective suppression of hydrogen transfer channels. Furthermore, non-covalent interactions of the optimized ground state geometries were investigated to identify intramolecular hydrogen bonds along which hydrogen transfer is most likely.

## Abstract 2

We present experimental evidence for soft X-ray induced intramolecular hydrogen transfer in the protonated synthetic tri-oligonucleotide *d*(^F^UAG) in the gas-phase (^F^U: fluorouracil).

## Graphical abstract

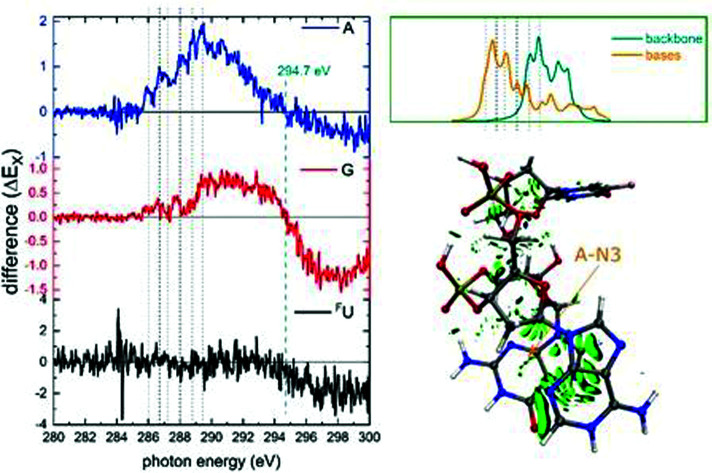

## 1 Introduction

Hydrogen bonding, one of the most important weak molecular interactions in nature, plays key roles for structure and dynamics of biomolecules such as proteins^1^ and DNA.^2^ Hydrogen bonds (H bonds) are for instance responsible for the secondary structure of proteins and DNA, with the DNA double helix being the most iconic example. H bonding is also one of the fundamental interactions in structural DNA nanotechnology.^3^ Because of their role in DNA structure, H bonds are often involved in dynamic processes. For instance, hydrogen transfer along intermolecular^4^ or intramolecular^5^ hydrogen bonds is an efficient way of energetic relaxation in DNA.

It is a well established fact that ππ* UV excitation of free nucleobases leads to population of excited states with very short intrinsic lifetimes ranging from sub-picosecond to several picoseconds.^6^ For instance, the de-excitation of cytosine can proceed radiationless on subpicosecond timescales *via* two consecutive conical intersections between the ππ* and nπ* states and the nπ* state and the ground state (S_0_).^7^ Such rapid decays can protect nucleobases from UV-induced radiation damage. Perun, Sobolewski and Domcke have theoretically investigated ultrafast radiationless decay in isolated adenine and identified similar 2-step processes, with the nπ* transition to the ground state proceeding *via* out of plane deformations of the 6-membered ring.^8^ This process appeared to be very sensitive to nucleobase bonding in DNA. Subsequent experiments indicated that for instance gas-phase adenosine has a significantly shorter excited-state lifetime as compared to adenine.^9^*Ab initio* calculations revealed that the femtosecond excited-state lifetime in adenosine is due to a barrierless excited-state deactivation mechanism, which involves proton transfer along the intramolecular O–H⋯N3 hydrogen bond between sugar and nucleobase through a conical intersection.^4^ Deexcitation mechanisms involving hydrogen transfer were also found for aminopyridine clusters, serving as model systems for Watson–Crick pairing in DNA:^5^ here, hydrogen transfer between two hydrogen-bonded aminopyridine rings proceeds by ultrafast internal conversion *via* a conical intersection which connects the electronic ground state and the excited ππ* charge transfer state.

As compared to UV excitation, inner shell excitation and ionization processes in DNA have received much less interest, despite their fundamental relevance for biological radiation damage. Therapeutic X-rays as well as fast ions used in radiotherapy efficiently induce inner shell excitations and ionizations, either directly in DNA or in surrounding water molecules. The resulting inner-shell vacancies are typically filled by Auger processes, leading to emission of energetic electrons which are of high relevance for radiation damage.^10^ Soft X-rays are an excellent tool to create such inner-shell ionization and excitation proocesses. In contrast to nucleobase ππ* or nπ* UV-excitation, resonant soft X-ray absorption in *e.g.* nucleobases implies 1s electron excitation to unoccupied valence orbitals (π*). Only subsequently, in an Auger-type decay process, one valence electron de-excites to fill the 1s vacancy while another valence electron is emitted. In most cases, the subsequent Auger decay will thus result in a doubly or even higher excited electronic configuration.

First X-ray absorption experiments^11^ on the doubly protonated gas-phase oligonucleotide [*d*GCAT + 2H]^2+^ found glycosidic bond-cleavage leading to formation of nucleobase ions (B^+^) and protonated nucleobase ions (BH^+^) as the main fragments. Glycosidic bond scission in a non-protonated oligonucleotide would in the first place produce (B–H) fragments. As a consequence, in ref. 11 the B^+^ fragment was ascribed to originate from a protonated nucleobase and the BH^+^ fragment would originate from a protonated nucleobase acting as an acceptor in a H transfer process. More recent experiments have challenged this interpretation, as even in deprotonated gas-phase oligonucleotides, soft X-ray absorption predominantly produces B^+^ and BH^+^ fragments.^10^ This finding suggests that B^+^ fragments can originate from non-protonated sites and their formation then normally involves single H transfer, while BH^+^ formation even requires double H transfer. To elucidate the underlying processes it is a straightforward strategy to measure the relative strength of single and double H-transfer processes in glycosidic bond-cleavage processes as a function of photon energy at all inner-shell absorption edges. The inner-shell edges of the elements constituting DNA are energetically well separated, which allows to selectively target inner shells in an element specific way. Furthermore, for a given element the inner-shell binding energies depend on the chemical environment. Resonant soft X-ray absorption is therefore sensitive to the local electronic structure. In a recent study, we have utilized soft X-ray spectroscopy at the N K-edge to investigate the influence of the protonation site on the electronic structure of protonated [*d*^F^UAG + H]^+^ at the nitrogen K-edge.^12^

In this work, we investigate [*d*^F^UAG + H]^+^ photofragmentation at the C, N, O and F K-edges. By means of near-edge soft X-ray absorption mass spectrometry (NEXAMS) spectroscopy^13,14^ partial ion yields of nucleobase fragments are determined. We do not focus on the photoabsorption process itself, but on the hydrogenation stages of the fragments to study H transfer and its enhancement or reduction by element and site-selective resonant soft X-ray absorption. The experimental quantity of interest will therefore be the relative intensity of BH^+^ with respect to B^+^.

## 2 Experiment

Two types of soft X-ray experiments were conducted to investigate DNA photofragmentation and photoabsorption in the gas phase. For both experiments, the modified single-stranded trinucleotides were purchased from LGC Biosearch Technologies. A 20 μM oligo solution in HPLC methanol was used. For 1 mL of solution, 20 μL acetic acid was added to facilitate protonation. The oligonucleotide cations were transferred from solution to the gas phase by means of electrospray ionization (ESI). Singly protonated [*d*(^F^UAG) + H]^+^ was then selected by a quadruple mass filter and guided to a radiofrequency (RF) ion trap. The stored oligonucleotide cations were then exposed to monochromatic soft X-rays. Eventually, the trap content was extracted into a reflectron-type time-of-flight mass spectrometer.

In the first type of experiments, a home-built tandem mass spectrometer^15,16^ was interfaced with the U49/2-PGM1 beamline^17^ of the BESSY II synchrotron (Helmholtz Zentrum Berlin, Germany). The mass-selected cations were trapped in a classical Paul trap and cooled down to room temperature by collisions with a pulse of helium buffer gas. The trapped protonated oligonucleotides where then exposed to monochromatic X-rays for a well-defined exposure time without buffer gas. After photoexposure, all positive ions in the trap were extracted into a high resolution 
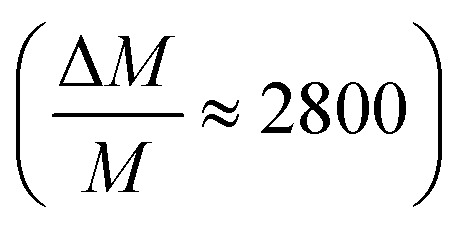
 time of flight (TOF) spectrometer. High resolution mass spectra were recorded for a number of well-defined X-ray energies. This experimental approach is optimized for high mass resolution and does not suppress fragmentation on long time scales.

The actual NEXAFS experiments were conducted with the ion trap apparatus,^18–20^ which is a fixed end-station at the high-resolution soft X-ray beamline UE52-PGM of the BESSY II synchrotron radiation facility. Here, oligonucleotide cation were accumulated in a cryogenic (*T* ≈ 20 K) linear RF trap. An action-spectroscopy approach was employed for determination of the soft X-ray photoabsorption cross sections, *i.e.* the photon energy *E*_X_ was ramped in small steps across the carbon, nitrogen, oxygen, and fluorine K-edge. For each *E*_X_, the trapped protonated oligonucleotides were exposed to monochromatic (Δ*E* ≈ 100 meV) soft X-rays and the entire trap content including the photo-products was extracted into a TOF mass spectrometer. The relative ion yields for the different photofragments as a function of *E*_X_ were then determined from the mass spectra. The much larger trapping volume of this apparatus allows for faster acquisition and is ideally suited for high resolution spectroscopy, requiring acquisition of hundreds of mass spectra per NEXAMS scan.

## 3 Calculations

The ground-state molecular structures in the gas phase were optimized using density functional theory (DFT) at the ωB97X-D/cc-pVDZ level. The minimum structure was confirmed by a Hessian calculation. The core-excitations were then computed using time-dependent DFT (TD-DFT) with Tamm–Dancoff approximation.^21^ Calculations were performed with restricted single excitation subspace that involved excitation only from the 1s orbital of a single atom at a time. The short-range exchange correlation functional SRC2-R1^22,23^ with the parameters *C*_SHF_ = 0.55, *μ*_SR_ = 0.69*a*_0_^−1^, *C*_LHF_ = 0.08 and *μ*_LR_ = 1.02*a*_0_^−1^ in combintation with a cc-pVDZ basis set was employed.

In this work, we computed the oscillator strenghts of 1s-excitations for C, N, O, and F atoms. Furthermore, starting from the optimized ground state equilibrium geometry of the *d*(^F^UAG)^+^ we determined the reduced density gradient for the system. Non-covalent interactions (NCI) were then visualized as isosurfaces of the reduced density gradient using the NCIPLOT method^24,25^ and the Visual Molecular Dynamics software package.^26^ All computations were done using the Q-Chem 5.1 quantum chemistry package.^27^

## 4 Results

**Fig. 1:**
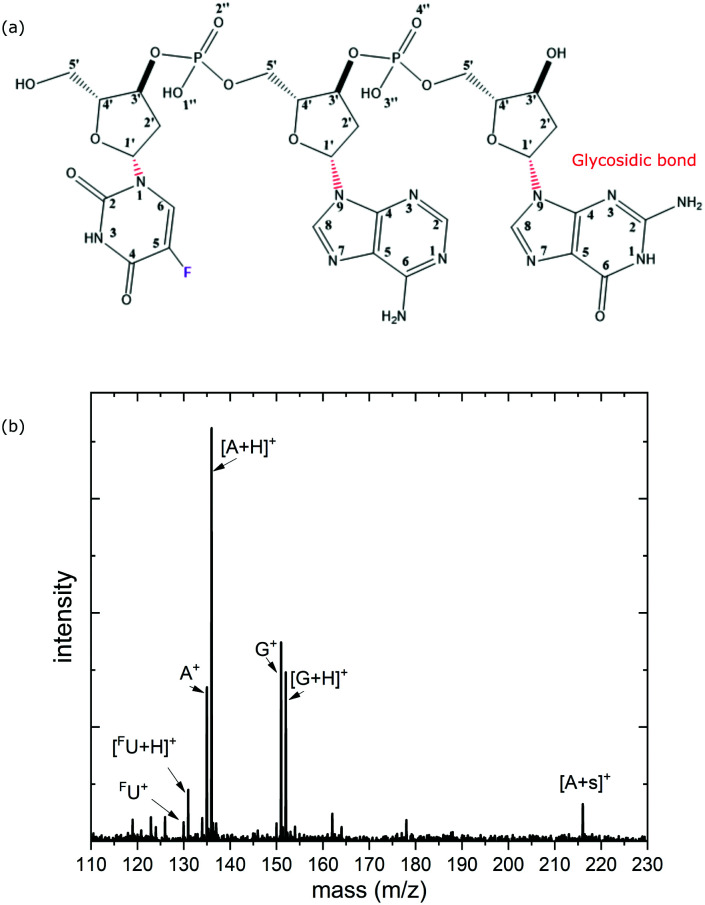
Soft X-ray photofragmentation mass spectrum of [*d*(^F^UAG) + H]^+^ (*m*/*z* 890) at *E*_X_ = 297.5 eV (carbon K-edge). (a) shows the molecular structure of *d*(^F^UAG) with the conventional atom numbering.

Fig. 1 shows a carbon K-edge mass spectrum of the soft X-ray photofragments of [*d*(^F^UAG) + H]^+^ for *E*_X_ = 297.5 eV at room temperature. Fragment ions with masses below 115 Da are not trapped under the experimental conditions of this study. Yields of fragments with *m*/*z* larger than 230 are very low at this photon energy. It is obvious that A^+^, [A + H]^+^, G^+^ and [G + H]^+^ fragments dominate the mass spectrum (in the following, we will often refer to B^+^ as B and BH^+^ as BH). Glycosidic bond cleavage is required for the generation of all these fragment ions. However, as obvious from mass spectrum in Fig. 1, the fragment ion yields related to A, G, and ^F^UH are very different, even though the nucleobases have identical abundances in the precursor molecule. Adenine and guanine related fragments are significantly more intense than those related to ^F^UH. The ratios of B and BH are also very different: AH is much stronger than A and also ^F^UH is much stronger than ^F^U, whereas G and GH are similar. The largest fragment observable in Fig. 1 is a commonly observed cyclic complex containing the five-membered sugar ring and the adenine nucleobase [A + s]^+^ (*m*/*z* 216).

Many weak additional fragments can be observed, some of which are due to further dissociation of the nucleobases. The fragmentation patterns are qualitatively similar in all soft X-ray mass spectra at the C, N, O, F K-edges.

### 4.1 Partial ion yield spectra

**Fig. 2:**
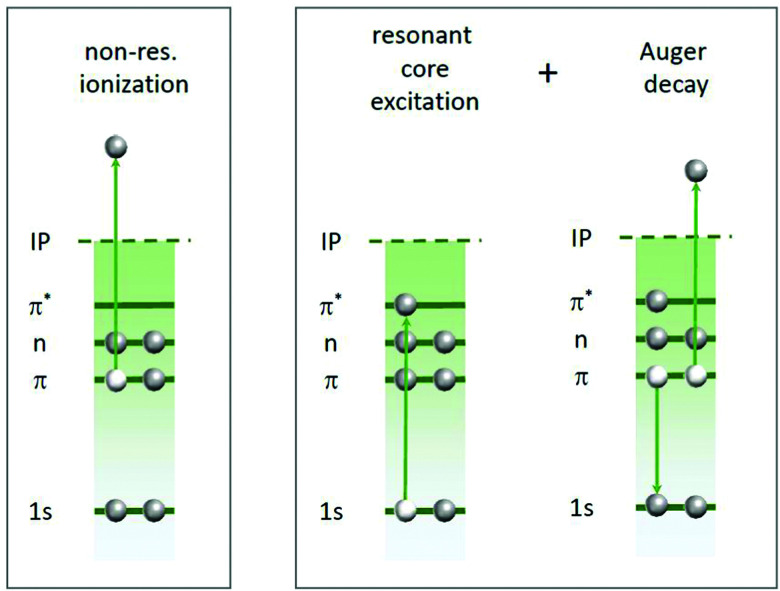
The two types of photoabsorption processes contributing to the experimentally obtained partial ion yields at a given inner-shell absorption edge. Left: Non-resonant ionization of a valence orbital (or a weaker bound core level). Right: Resonant inner-shell excitation into unoccupied molecular orbitals followed by Auger decay. The Auger decay can involve various combinations of valence electrons.

In the following, we report the yields of the main 6 photofragment ions B^+^ and BH^+^ as a function of photon energy *E*_X_. We refer to these spectra as NEXAMS^13^ spectra. In the soft X-ray range, valence photoabsorption cross-sections (and thus also partial photofragment ion yields) decrease with increasing photon energy. At the C, N, O, F K-edges however, resonant excitation and ionization of the respective inner shells leads to increasing photoabsorption cross sections. For better comparison of the NEXAMS spectra, we normalize to the (continuous) background signal which results from non-resonant photoionization of valence electrons or weaker bound core electrons (see Fig. 2). The normalized partial ion yield *Y*^norm^ is obtained from the raw partial ion yield *Y* as the following:1
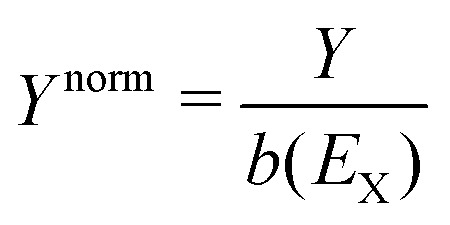
where

**Fig. 3:**
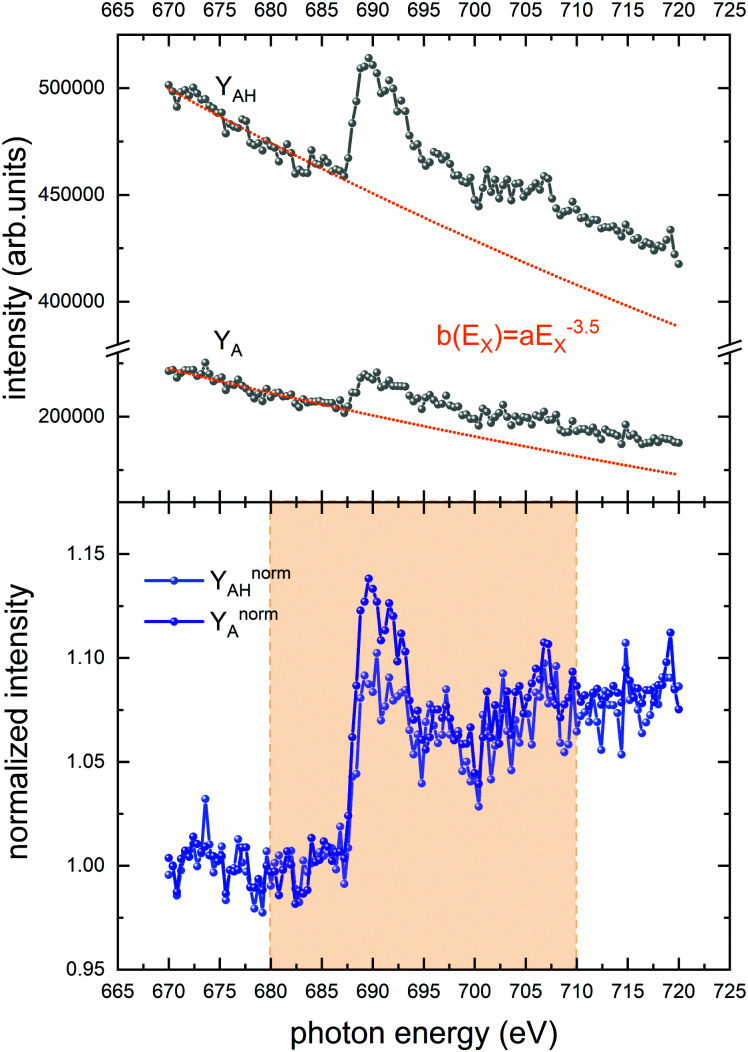
Normalization for AH^+^ and A^+^. Top panel: raw data and fitted baseline function *b* (*E*_X_). Bottom panel: normalized data. The yellow area is our region of interest and covers the resonant spectral features.

2 *b*(*E*_X_) = *aE*_X_^−3.5^

is a baseline function fitted to the pre-edge data of *Y*, which represents the background with *E*_X_^−3.5^ being the common dependence of the cross section for valence photoionization on the photon energy *E*_X_. *a* is a parameter defined by the pre-edge data. For each nucleobase B^+^, we have *Y*^norm^_B_, *Y*_B_, and *b*_B_ (*E*_X_); for each protonated nucleobase BH^+^, we have *Y*^norm^_BH_, *Y*_BH_, and *b*_BH_(*E*_X_). An example for the baseline determination of the spectra at F K-edge is shown in the Fig. 3, where the partial ion yields for A^+^ and AH^+^ are used as an example. For most of the edges, the typical structure of NEXAMS spectra is observed: The low energy part of the spectrum features resonances, which are due to inner-shell excitations into unoccupied molecular orbitals (see Fig. 2), usually of π* character. At intermediate energies, higher lying excited states are involved. A multitude of energetically close transitions makes them difficult to assign. The high energy part is due to inner-shell ionizations into the continuum and lacks resonant features.

#### 4.1.1 Carbon K-edge

**Fig. 4:**
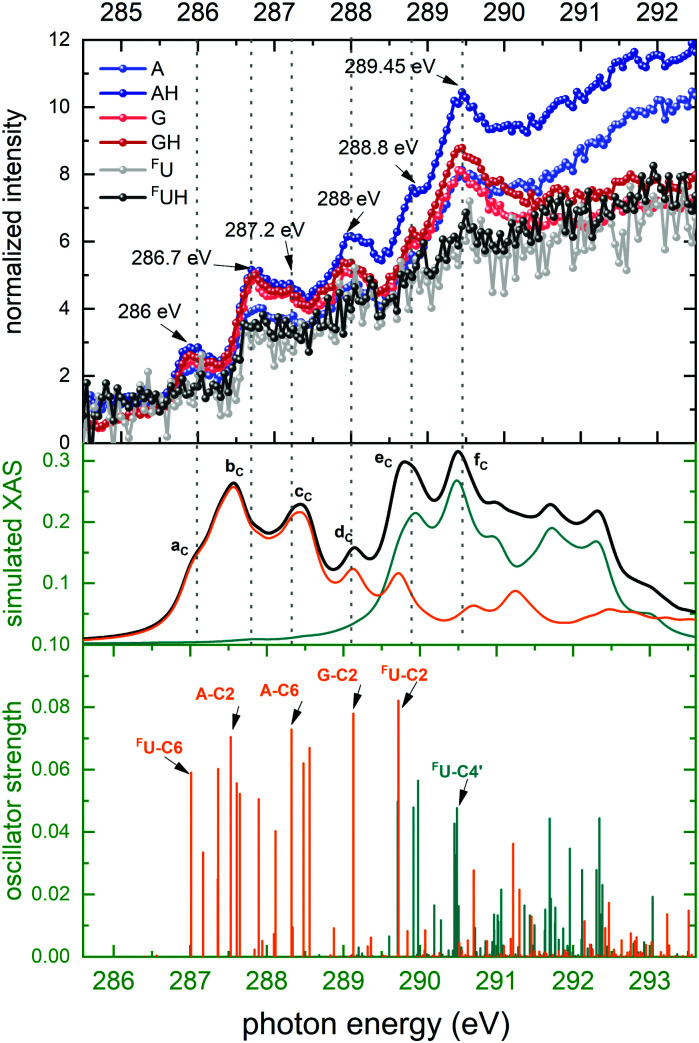
Top panel: Normalized Carbon K-edge partial ion yields *Y*^norm^ for [*d*(^F^UAG) + H]^+^ for nucleobase fragments (A^+^, G^+^ and ^F^U^+^) and their singly protonated counterparts (AH^+^, GH^+^, ^F^UH^+^). Bottom panel: Stick spectrum of TD-DFT oscillator strengths for C 1s transitions. Core excitations localized on the nucleobases are colored orange, while core excitations on the backbone are colored green. 6 particular transitions are labeled. Middle panel: TD-DFT absorption spectrum obtained by convoluting the stick spectrum with Gaussian functions of fwhm of 0.4 eV. The black curve is the sum spectrum of all core transitions based either on the nucleobases (orange) or on the backbone (green). Note that for almost all edges, theoretical and experimental data exhibit a small relative energy shift (here: about 1 eV). Here and in the following figures, slightly shifted energy sections are therefore presented for theory and experiment, in order to match peak positions.

Fig. 4 shows experimental and theoretical photon absorption results at the C K-edge of [*d*(^F^UAG) + H]^+^. Six resonant peaks at 286 eV, 286.7 eV, 287.2 eV, 288 eV, 288.8 eV and 289.45 eV can be identified in the partial ion yield spectra. The overall spectral appearance is qualitatively similar for all BH^+^ and B^+^ ions, though for ^F^U^+^ and ^F^UH^+^, the low intensities make it more difficult to distinguish individual resonance peaks.

The corresponding TD-DFT oscillator strengths are shown in the bottom panel of Fig. 4 as a stick spectrum. For better comparison to the experiment, the middle panel of Fig. 4 shows the theoretical data convoluted with a Gaussian of full width half maximum (fwhm) of 0.4 eV. This peak broadening reflects the fact that a given electronic transition can involve a broad range of vibrational levels in the final state. The used fwhm gives good agreement to the experimental data.

The convoluted TD-DFT absorption spectrum exhibits the same 6 maxima, labeled as *a*_C_ to *f*_C_, as found in the experimental data. The color code on the stick spectra reveals that all transitions below about 290 eV are localized on the nucleobases which corresponds to the first 4 peaks in the experimental data. Bands *e*_C_ and *f*_C_ both have mixed character and thus contain contributions from transitions on nucleobases and backbone. We have selected six transitions with high oscillator strength to represent each maximum. The absorption bands *a*_C_ to *e*_C_ have π* character, while the *f*_C_ band is of σ* character.

#### 4.1.2 Nitrogen K-edge

**Fig. 5:**
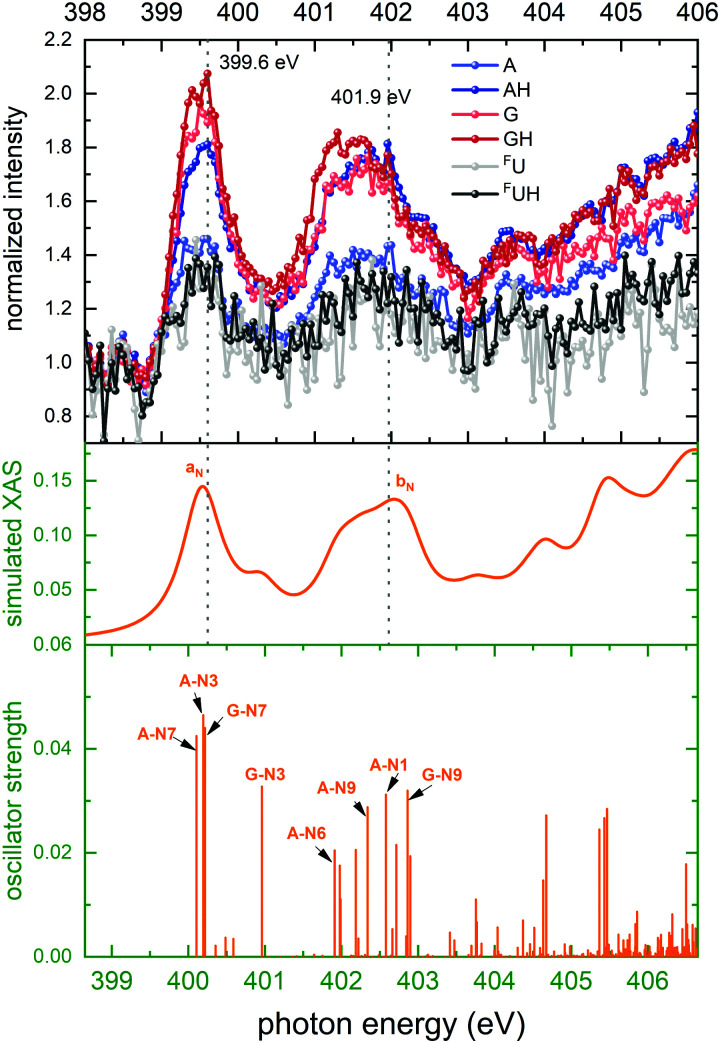
Top panel: Normalized ion yields of A^+^, G^+^, and ^F^U^+^ and AH^+^, GH^+^, and ^F^UH^+^ following X ray absorption near the N K edge by [*d*(^F^UAG) + H]^+^. Bottom panel: Stick spectrum of TD-DFT oscillator strengths for N 1s transitions. Middle panel: Convoluted TD-DFT data (Gaussian fwhm = 0.6 eV). Color codes as in Fig. 4.

The N K-edge NEXAMS nucleobase spectra of [*d*(^F^UAG) + H]^+^ are shown in the top panel of Fig. 5. This spectrum is dominated by two absorption bands at 399.5 eV and 401.5 eV. It is to be noted that the ion spectra of A (AH) and G (GH) in Fig. 5 have different relative intensities at the two bands. The intensities for G and GH are higher in the first band than in the second one, while for A and AH the intensities are quite similar.

The TD-DFT X-ray absorption spectrum shows the same two absorption bands, labeled *a*_N_ and *b*_N_. *a*_N_ is solely due to 1s excitation of the imine N atoms (A-N7, A-N3 and G-N7) in adenine and guanine. The imine A-N1 atom of adenine is the most likely protonation site in [*d*(^F^UAG) + H]^+^. When A-N1 is protonated, the lowest energy absorption transition localized at A contributes to the *b*_N_ band rather than to the *a*_N_ band.^12^ Band *b*_N_ is generally comprising transitions on amine nitrogen atoms in all three nucleobases. The final states of the transitions contributing to both bands *a*_N_ and *b*_N_ all have π* character and are localized on the nucleobases. This is discussed in detail in ref. 12.

#### 4.1.3 Oxygen K-edge

**Fig. 6:**
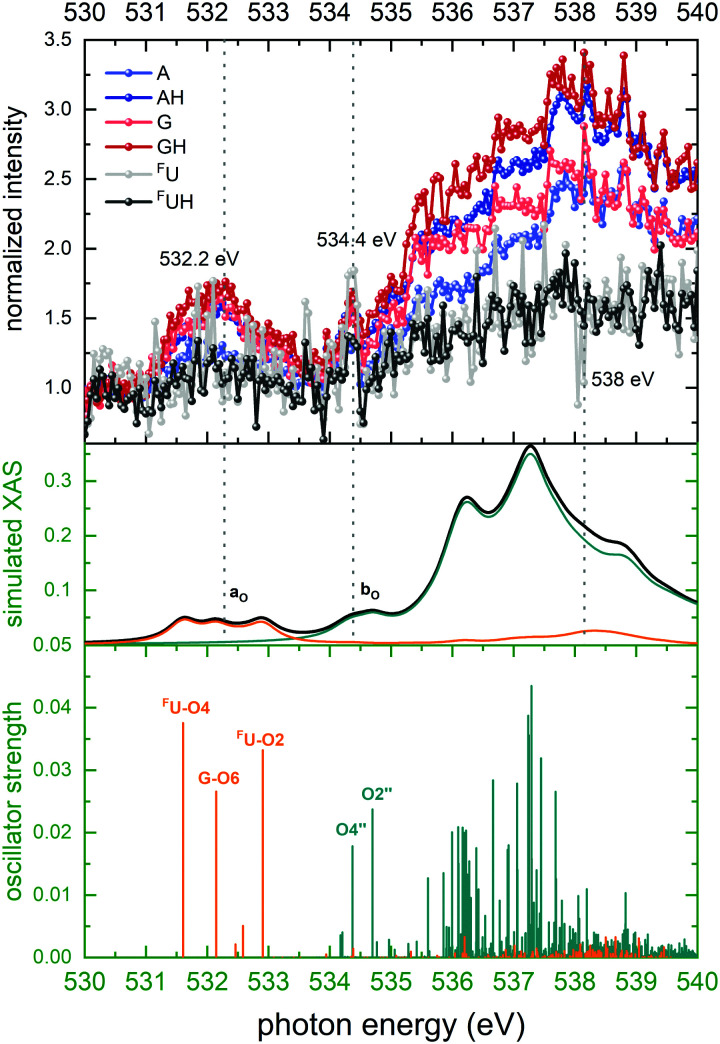
Top panel: Normalized ion yields of A^+^, G^+^, and ^F^U^+^ and AH^+^, GH^+^, and ^F^UH^+^ following X ray absorption near the O K edge by [*d*(^F^UAG) + H]^+^. Bottom panel: Stick spectrum of TD-DFT oscillator strengths for O 1s transitions. Middle panel: Convoluted TD-DFT data (Gaussian fwhm = 0.4 eV). Color codes as in Fig. 4.

The O K-edge NEXAMS spectra are shown in the top panel of Fig. 6. This spectrum features two relatively broad absorption bands at energies of 532.2 and 538 eV. Furthermore, a weak band at an energy of 534.4 eV is observed. The TD-DFT X-ray absorption spectrum has similar absorption bands as the experimental data. Band *a*_O_ corresponding to the peak at 532.2 eV consists of 1s-π* transitions of the O atoms in ^F^U and guanine. Band *b*_O_ is based on the backbone and is due to core transitions in the O2′′ and O4′′ atoms, which are double bonded with P atoms in the phosphate group. The TD-DFT calculation shows that the broad featureless high-energy part of the spectrum (above 535 eV) is mostly due to a large number of energetically closely-spaced 1s excitations localized on backbone sugar-phosphate sites.

#### 4.1.4 Fluorine K-edge

**Fig. 7:**
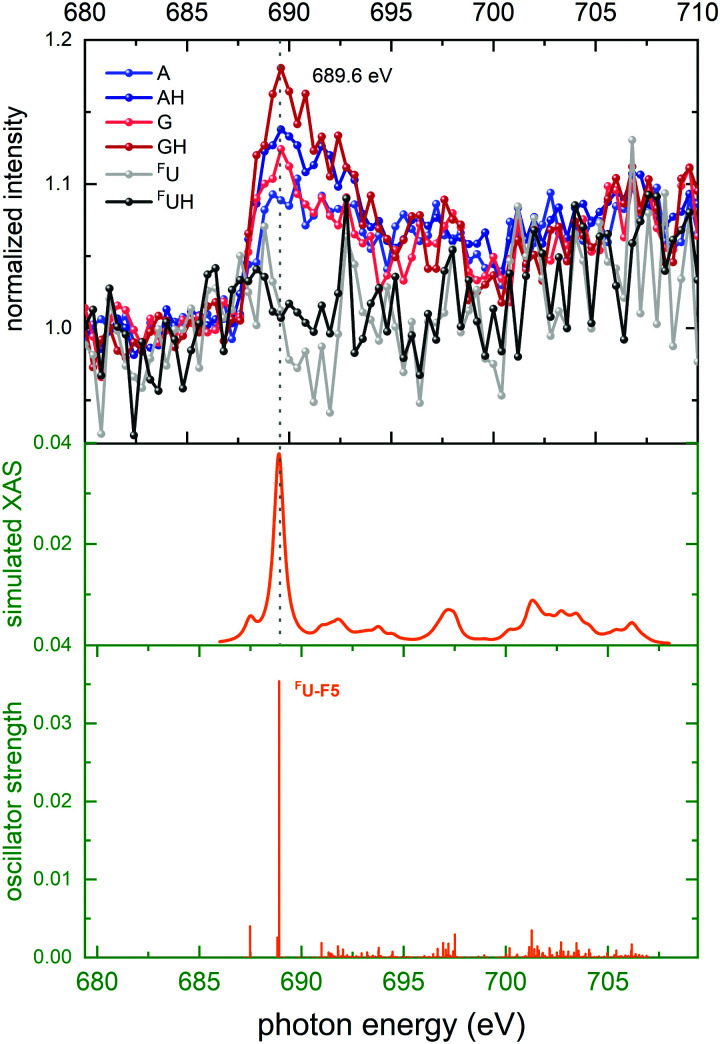
Top panel: Top panel: Normalized ion yields of A^+^, G^+^, and ^F^U^+^ and AH^+^, GH^+^, and ^F^UH^+^ following X ray absorption near the F K edge by [*d*(^F^UAG) + H]^+^. Bottom panel: Stick spectrum of TD-DFT oscillator strengths for F 1s transitions. Middle panel: Convoluted TD-DFT data (Gaussian fwhm = 0.6 eV). Color codes as in Fig. 4.

Fig. 7 (top panel) shows the last of the K-edge NEXAMS spectra for [*d*(^F^UAG) + H]^+^, the ones taken in the F K edge. Note that the entire molecule contains only a single F atom, located in the halogenated nucleobase ^F^U. Accordingly, the cross section for F 1s absorption is very small as compared to C (29 atoms), O (16 atoms), and N (12 atoms). As a consequence, the F K-edge spectra have relatively low intensity above the pre-edge spectra, nevertheless a broad peak at 689.6 eV is observed which can be ascribed to the ^F^U-F5 transition. It is interesting to note that this peak is only observed for nucleobase fragment ions other than ^F^U^+^ and ^F^UH^+^. ^F^U is a single-ring pyrimidine while the purines A and G consist of two rings. Therefore it is possible that photoabsorption in ^F^U is more prone to fragment the pyrimidine ring and as a consequence to an absence of the fluorine resonance in the ^F^U^+^ spectrum.

In the theoretical data (Fig. 7 bottom panel) the stick spectrum shows that only a single transition to the σ* orbital is dominating. In contrast to the strong lowest energy 1sπ* transitions observed at the other edges, here the final σ* state is distributed not just over the excited nucleobase but also over the entire sugar-phosphate backbone and it is energetically above the π* states on A and G.

## 5 Discussion

The spectral shapes of background subtracted, normalized ion yields of nucleobase fragments following soft X-ray photoabsorption by *d*(^F^UAG) are in excellent agreement with our TD-DFT calculations. The overall shape of the partial ion yield spectra for all B^+^ and BH^+^ ions are similar. However, in particular in the spectral regions of resonant absorption features there are distinct differences in the peak intensities between nucleobase (B^+^) and protonated nucleobase (BH^+^) fragment ions. The photon energy dependence of relative differences in B^+^ and BH^+^ may thus shed light on the processes that define the extent of H transfer accompanying glycosidic bond cleavage induced by soft X-ray K shell excitation.

Important information for identification of different H transfer mechanisms can be drawn from the site-selectivity (X-ray absorption in specific nucleobases *versus* absorption in the backbone) of soft X-ray absorption at specific resonances. For photon energies near the C K edge, 29 C atoms contribute to X-ray absorption of which 15 are backbone constituents and 14 are nucleobase constituents. As Fig. 4 shows the nucleobase C atoms (orange sticks/spectra in the theoretical data) are primarily addressed at energies below 290 eV, while above 290 eV photon absorption in backbone C atoms (green sticks/spectra in the theoretical data) dominates. The situation for O is similar to the C case, with the exception that the two distinct bands are observed. The band centered at 532 eV is due to nucleobase excitation and the broader band at higher energy is dominated by backbone transitions (see Fig. 6, same color coding as for C). For both, N atoms and F atom, the situation is less complicated as both elements are solely found in the nucleobases, and photoabsorption near the N and F edges must therefore be localized at the nucleobases.

For a better visualization of the enhancement or reduction of H transfer at element-specific and site-specific soft-X-ray K-shell absorption resonances we introduce difference spectra for the nucleobases. These spectra show the difference Δ(*E*_X_) in ion yield between protonated and radical nucleobase ions BH^+^ and B^+^ as a function of X-ray energy.

### 5.1 Difference spectra

Δ(*E*_X_) is defined as follows:

3a Δ(*E*_X_) = *Y*^norm^_BH_(*E*_X_) − *Y*^norm^_B_(*E*_X_)

3b
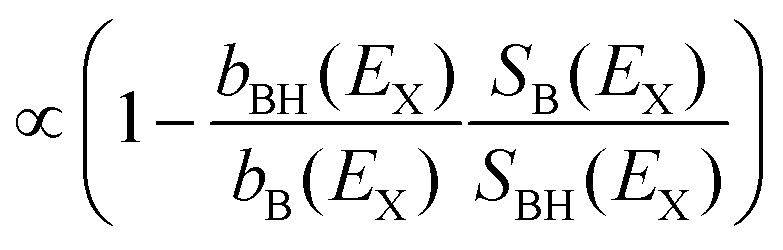
where *S*_*i*_(*E*_X_) is the total intensity on top of the non-resonant background (*S*_*i*_ = (*Y*^norm^_*i*_(*E*_X_) − 1)*b*_*i*_(*E*_X_) with *i* being either B^+^ or BH^+^. The Δ(*E*_X_) spectra therefore start at zero in the pre absorption edge region. The *b*_BH_(*E*_X_)/*b*_B_(*E*_X_) = *a*_BH_/*a*_B_ is constant (see eqn (2)) and quantifies the ratio of the pre-edge intensities of BH^+^ and B^+^.

Table 1 displays the pre-edge ratios *a*_BH_/*a*_B_ for the K-edges of C, N, O, and F. These ratios quantify the transfer of one or more H atoms during glycosidic bond cleavage triggered by non-resonant soft X-ray absorption involving valence electrons or weaker bound inner-shell electrons. Lowest ratios of approximately 1.1 are found for G. Slightly higher (10–30%) values are observed for ^F^U. The pre edge ratios for A are about twice as high as the ones for G and ^F^U. Most likely, the ratios for A are systematically higher because A is the preferred protonation site in [*d*(^F^UAG) + H]^+^.^12^ Therefore AH formation just requires single H transfer, while the transfer of two atoms is needed to produce ^F^UH and GH.

**Table 1:** Pre-edge ratios (*a*_BH_/*a*_B_) of A, G, and ^F^U for the C, N, O, and F K-edges

K-edge	*a*_AH_/*a*_A_	*a*_GH_/*a*_G_	*a* ^F^UH/*a*^F^U
C	2.92	1.26	1.68
N	2.21	1.21	1.42
O	2.33	1.13	1.39
F	2.25	1.11	1.21

For soft X-ray photon energies where Δ(*E*_X_) is positive, *S*_BH_(*E*_X_)/*S*_B_(*E*_X_) is larger than the pre-edge ratio *a*_BH_/*a*_B_, *i.e.* H transfer to the nucleobase must be more efficient. Negative Δ(*E*_X_) values indicate less efficient H transfer.

**Fig. 8:**
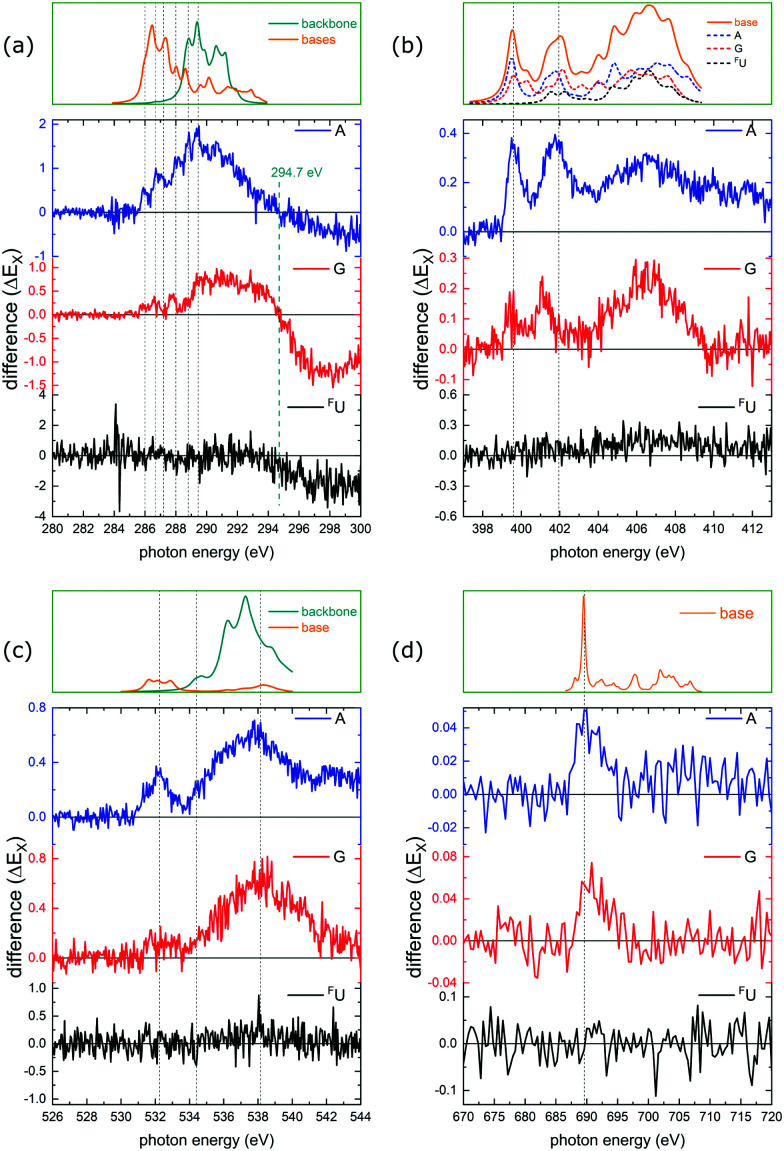
Difference spectra Δ(*E*_X_) at the K-edges of (a) C, (b) N, (c) O, and (d) F. The vertical dotted gray lines indicate the resonant photon absorption maxima observed in Fig. 4–7. Color codes as in Fig. 4(a, c and d). In (b) nucleobases are shown separately.

The full set of Δ(*E*_X_) difference spectra at the C, N, O, and F K-edges is plotted in Fig. 8. Only at the C K-edge, Δ(*E*_X_) features negative values for photon energies above 294.7 eV (photoionization regime). All other Δ(*E*_X_) spectra are positive with maxima located at the photon energies matching the absorption resonances. The top panel for each K-edge displays the convoluted TD-DFT data with the usual color coding (nucleobase transitions: orange, backbone transitions: green). For the N K-edge, the TD-DFT data is shown for the individual nucleobases.

The general conclusion from these findings is that resonant K-shell excitation of soft X-rays promotes H transfer. In the following we will focus on a more detailed investigation of the photoexcitation regime for the nucleobases adenine and guanine. Due to the single absorption site, statistics for ^F^U are insufficient for a meaningful analysis of the respective data (see Fig. 8). For adenine and guanine the trends observed in the difference spectra can be summarized as follows:

Adenine: for A, the Δ_A_(*E*_X_) spectra qualitatively agree with the respective partial ion yield spectra for all K-edges, *i.e.* the X-ray induced facilitation of H transfer is not site specific and is observed for nucleobase and backbone absorption.

Guanine: for G, Δ_G_(*E*_X_) shows a clear site-dependence. At the C K-edge, nucleobase absorption has almost no effect on H transfer, whereas backbone absorption does. At the N K-edge, both low energy resonances are reduced to their low-energy flank which is caused by absorption on the A nucleobase (see TD-DFT data in the top panel). At the O K-edge, the nucleobase resonance at 532 eV is almost absent (note, that only G and ^F^U feature O atoms), whereas the backbone feature at higher energies is similar to the A case.

The observed Δ(*E*_X_) trends are summarized in Table 2, where the upwards arrows mark an increasing ratio while the “—” indicates no change in intensity.

**Table 2:** Classification of Δ(*E*_X_) for resonant photoexcitation on different parts of the [*d*(^F^UAG) + H]^+^ molecule. The upwards arrows mark an increasing ratio while the “—” indicates no change in intensity

Site	AH^+^/A^+^	GH^+^/G^+^
A	↑	↑
G	↑	—
^F^U	↑	↑
Backbone	↑	↑

### 5.2 Hydrogen transfer pathways in glycosidic bond cleavage

In the last section we demonstrated clear evidence for a direct effect of resonant X-ray absorption on H-transfer accompanying glycosidic bond cleavage (see Fig. 8). Interestingly, [B–H]^+^ fragments resulting from photoionization of non-protonated bases without subsequent H-transfer are very much absent in the spectra, *i.e.* glycosidic bond cleavage without H transfer is very unlikely, at least for nucleobases which were not initially protonated. The only observed channel that does not involve H-transfer is formation of B^+^ from an initially protonated nucleobase, most likely A. We therefore limit this discussion to the pathways yielding B^+^ and BH^+^ with a focus on the absorption site-dependent enhancement of BH^+^ formation.

**Fig. 9:**
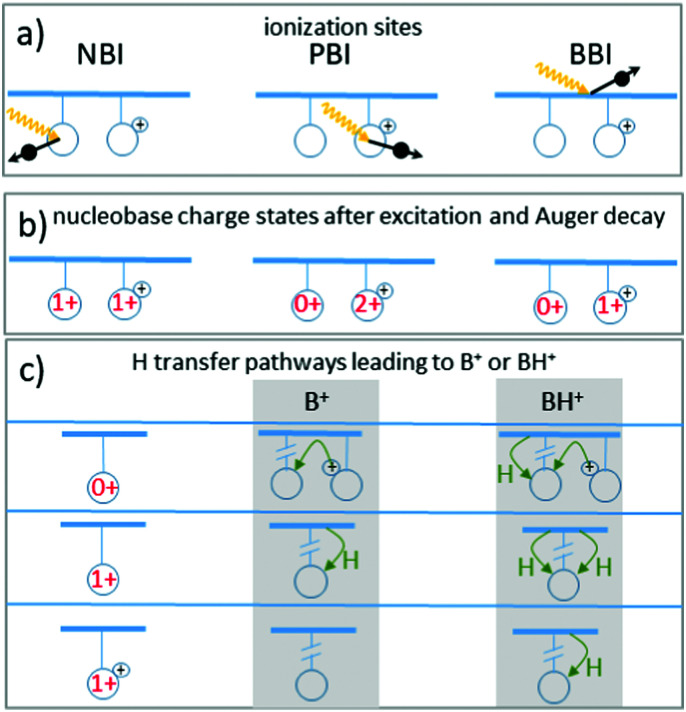
Schematics of the pathways leading to B^+^ or BH^+^ fragments after soft-X-ray K-shell excitation and subsequent Auger decay in [*d*(^F^UAG) + H]^+^. (a) Sketch of the three different photoionization sites: neutral base ionization (NBI), protonated base ionization (PBI) and backbone ionization (BBI). (b) Nucleobase charge states after photoionizaton for the three sites. (c) Nucleobase charges after charge equilibration and subsequently required H transfer processes for formation of either B^+^ or BH^+^. The black “+”-sign indicates the existing initial charge state due to protonation, while charge states in red indicate the total charge on the nucleobase.

For light atoms such as C, N and O, K-shell vacancies as induced by X-ray absorption are predominantly filled non-radiatively by Auger decay (see Fig. 2, right panel). As the Auger decay process is localized at the site of the K-vacancy and leads to an ionization at this site, in the following we will refer to inner-shell photoexcitation plus Auger decay as X-ray photoionization. The process can be classified according to the photoionization site into backbone based ionization (BBI), neutral nucleobase ionization (NBI) or protonated nucleobase ionization (PBI) (see Fig. 9a).

The intermediate [*d*(^F^UAG) + H]^2+^ resulting from photoionization can leave nucleobases uncharged, singly charged and doubly charged (see Fig. 9b). The resulting charge distribution is unlikely to be energetically favorable. PBI will even lead to two positive charges localized on a single nucleobase. It is likely that charge migration and relaxation proceeds on shorter timescales as compared to fragmentation. Glycosidic bond cleavage will then occur as a subsequent step, involving either a neutral nucleobase, a singly ionized nucleobase or a protonated nucleobase. Fig. 9c shows the H transfer process(es) that are required for the formation of B^+^ and BH^+^ fragments for the three different cases.

Cleavage of the glycosidic bond between sugar and nucleobase is one of the most common dissociation processes in oligonucleotides. Wu *et al.* have recently studied glycosidic bond cleavage in N7-protonated deoxyguanosine and deoxyguanosinemonophosphate and in N1-protonated deoxyadenosine and adenenosinemonophosphate^28^ using collision-induced dissociation (CID) and DFT. They observed a multi-step dissociation process, where bond scission is accompanied by nucleobase rotation and transfer of the deoxyribose 2' H atom to the G-N9 or to the A-N9 site, respectively occuring before the actual cleavage process. For the case of [*d*(^F^UAG) + H]^+^, this explains that with A being the most likely protonation site, the A pre-edge ratio *a*_AH_/*a*_A_ is systematically higher than the pre-edge ratios for the other two nucleobases (see Table 1). We therefore consider glycosidic bond cleavage accompanied by H-transfer from the sugar (*i.e.* from the backbone) the generic H transfer process for the system under study (H transfer process I). H transfer can also occur along intramolecular H bonds. This process has been intensively studied as a potential efficient ultrafast decay channel in excited nucleosides. For example, it has been shown that excited state lifetimes of nucleosides are significantly shorter than those of the respective isolated nucleobases.^4,8^ In both systems electronic excitations decay radiationless and this decay typically proceeds *via* a conical intersection between the low-lying excited states and the ground state. The short lifetime for the nucleoside was attributed to hydrogen transfer processes along the nucleobase-sugar hydrogen bonds. We will show in the following, that H transfer along intramolecular hydrogen bonds (H transfer process II) very likely is responsible for the pronounced dependence of H transfer on X-ray photon energy.

Note that H atoms could also be transferred from one nucleobase to another. In the following, however, we focus on H from the sugar-phosphate backbone, where the nucleobase acts as H acceptor, and the backbone as H donor. The bottom panel of Fig. 9 gives examples for H and proton transfer processes accompanying glycosidic bond cleavage, that can lead to the observed B^+^ and BH^+^ cations from a neutral, singly positive and doubly positive/protonated nucleobase moiety.

**Fig. 10:**
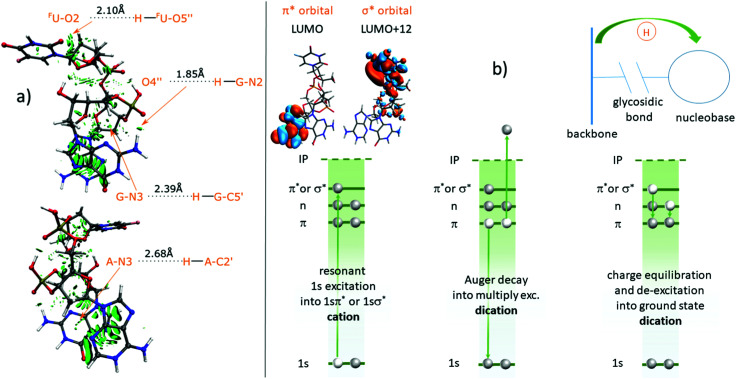
(a) NCI analysis of [*d*(^F^UAG) + H]^+^. Rotate the top structure by 180 degree to get the bottom structure. The NCI surfaces correspond to *s* = 0.5 au and a color scale of −0.03 < *ρ* < 0.03 a.u. Four nucleobases related hydrogen bonds are found. The bond lengths are indicated. (b) Multistep-scenario for ionization and H transfer along H bonds.

A first step for a deeper investigation of H transfer along intramolecular H bonds is the identification of such bonds in [*d*(^F^UAG) + H]^+^. In our previous work,^12^ we have already theoretically studied *d*(^F^UAG) as a neutral system as well as in A-N1 protonated and in G-N7 protonated form. Non-covalent interactions (NCI) are characterized by a combination of large electron density gradient at low electron density. As a consequence, the quotient of density gradient and density, *i.e.* the reduced density gradient, becomes large in NCI regions.^24^Fig. 10a shows the reduced density gradient for A-N1 protonated *d*(^F^UAG)^+^ in the form of isosurface (0.5 a.u.). The most prominent NCI is the strong NCI due to π-stacking of A and G. However, a close inspection of the more subtle features of Fig. 10a reveals four localized H bonds between nucleobase and backbone (see arrows), which are present in the neutral molecule as well as for both protonation sites. The three most obvious hydrogen acceptors that can be identified in the nucleobases are ^F^U-O2, A-N3 and G-N3. Interestingly, G also has a H bond donor site at G-N2, which binds to the O4′′ in the phosphate group.

Fig. 10b shows a sketch of the proposed soft X-ray absorption induced scenario. 1s photoexcitation (*e.g.* to a π* orbital) leads to formation of a core excited *d*(^F^UAG)^+^1sπ* or 1sσ*. The induced K-shell vacancy has a typical lifetime of few femtoseconds only^29^ and will predominantly be filled by an Auger decay. The intermediate state formed by this Auger decay is usually a multiply-excited dication state, with two valence holes and an electron in the π* orbital. This is different from *e.g.* UV photoexcitation, which leads to singly excited states (*e.g.* ππ* or πσ*) and no ionization. In a last step, the multiply excited dication further decays to the ground state and this process is accompanied by H transfer along an H bond. Glycosidic bond cleaveage is occuring when the H transfer is complete, implying that the H transfer process needs to be very fast.

Without detection of the emitted Auger electron, the Auger decay can involve any combination of valence electrons and accordingly it can lead to a multitude of multiply excited states.^30^ However, the experimental data suggests that H transfer is a very general phenomenon. This suggests that H transfer is part of the de-excitation pathway for most intermediate multiply excited configurations. The H transfer could possibly be due to radiationless rearrangement processes proceeding *via* conical intersections, as those observed for nucleobases and nucleotides previously.^8,9^

How does the observed absorption site dependence fit to this scenario? From Fig. 8 we have already concluded that H-transfer to adenine pretty much agrees with the partial ion yield spectra for all four absorption edges under study. This implies that X-ray absorption unspecifically facilitates H transfer to A. For G we observed a strong selectivity: H transfer to G is only facilitated when absorption is not occurring on the G. In our previous publication we have already shown that partial ion yields for G^+^ and GH^+^ are reduced for photoabsorption on G.^12^ We have explained this observation by fast fragmentation of the photoexcited G, before the excitation energy can be distributed over the entire molecule. The inverse site effect on H transfer shows that for those G moieties not destroyed by direct photoabsorption, the photoabsorption process hampers H-transfer to the base, possibly because of faster glycosidic bond cleavage. The reason why this effect is unobserved for A could lie in the fact that A is the pronounced protonation site and formation of AH^+^ just requires single H transfer, whereas formation of GH^+^ requires double H transfer.

## 6 Conclusion

In this article, we have investigated the influence of resonant soft X-ray absorption near the K-edges of C, N, O and F on H transfer towards the nucleobases. Photoinduced glycosidic bond cleavage was found to always lead to formation of B^+^ or BH^+^ fragment ions, even though mere bond scission would lead to formation of (B–H)^+^. H transfer towards the nucleobases is thus a very fundamental phenomenon. By investigation of the ratio between BH^+^ yields and B^+^ yields as a function of photon energy, we have quantified the influence of soft X-ray photon energy on the H transfer process. For A, we do not observe a pronounced site sensitivity and the probability for H transfer depends on X-ray energy in the same way the partial ion yield of A^+^ does. For G on the other hand, H transfer appears to be quenched for soft X-ray absorption on the G-moiety. Possibly, for direct ionization of the G nucleobase, due to the high excitation energy glycosidic bond cleavage is completed before H-transfer is.

The actual H transfer process is difficult to disentangle in this experiment, as inner-shell excitation of the precursor cation is quickly followed by an Auger decay that leads to a dication that can be in a multitude of different multiply excited states. It is striking that despite the large variety in intermediate excited states, H transfer is such a robust feature that for A does not even depend on the absorption site and final state. For UV photoexcitation of nucleobases and nucleotides, it has previously been found that de-excitation often proceeds radiationless and ultrafast *via* a conical intersection to a H transfer state.^4^ It is feasible that also in resonant soft X-ray excitation, similar ultrafast H-transfer processes are at play and we have used TD-DFT to identify the intramolecular H bonds, along which such transfer could proceed. For a deeper understanding of the underlying dynamics, future studies with coincident detection of the Auger electrons and/or time-resolved studies would be very helpful.

## Conflicts of interest

There are no conflicts to declare.

## Supplementary Material

CP-024-D1CP05741C-s001
