# Supplementary material for: Intramolecular hydrogen transfer in DNA induced by site-selective resonant core excitation
Source: Phys Chem Chem Phys. 2022 Mar 9;24(13):7815–25. doi: 10.1039/d1cp05741c (PMC8966622; doi:10.1039/d1cp05741c)
Supplement: CP-024-D1CP05741C-s001 [file CP-024-D1CP05741C-s001.pdf]

Electronic Supplementary Material (ESI) for Physical Chemistry Chemical Physics.
This journal is © the Owner Societies 2022

Supplementary information: Intramolecular hydrogen transfer in DNA induced by site-selective resonant core excitation

December 16, 2021

Table 1: Transition analysis at the carbon K-edge. Excitation energies are given in eV. f is the oscillator strength and L is the lowest unoccupied molecular orbital (LUMO). Only transitions with the high amplitudes are given.

excitation center	excitation energy (f)	transition (amplitude)
$FU-C6$	287.01 (0.059)	$1s \rightarrow$ L+2 (0.97)
$A-C5$	287.17 (0.033)	$1s \rightarrow$ L+1 (0.91)
$A-C8$	287.36 (0.06)	$1s \rightarrow$ L+1 (0.61)
$A-C2$	287.53 (0.07)	$1s \rightarrow$ L+1 (0.94)
$A-C4$	287.61 (0.056)	$1s \rightarrow$ L+0 (0.87)
$G-C8$	287.65 (0.052)	$1s \rightarrow$ L+6 (0.63)
$G-C4$	287.9 (0.051)	$1s \rightarrow$ L+3 (0.63)
$FU-C5$	288.11 (0.04)	$1s \rightarrow$ L+2 (0.92)
$A-C6$	288.32 (0.073)	$1s \rightarrow$ L+0 (0.94)
$FU-C4$	288.48 (0.062)	$1s \rightarrow$ L+2 (0.88)
$G-C6$	288.56 (0.067)	$1s \rightarrow$ L+3 (0.83)
$G-C2$	289.13 (0.078)	$1s \rightarrow$ L+6 (0.83)
$G-C5'$	289.71 (0.05)	$1s \rightarrow$ L+11 (0.58)
$FU-C2$	289.72 (0.082)	$1s \rightarrow$ L+5 (0.95)
$A-C5'$	289.91 (0.048)	$1s \rightarrow$ L+7 (0.77)
$FU-C3'$	289.97 (0.056)	$1s \rightarrow$ L+7 (0.7)
$A-C4'$	290.45 (0.043)	$1s \rightarrow$ L+13 (0.59)
$A-C3'$	290.47 (0.033)	$1s \rightarrow$ L+11 (0.46)
$FU-C4'$	290.48 (0.048)	$1s \rightarrow$ L+12 (0.34)
$FU-C6$	290.7 (0.028)	$1s \rightarrow$ L+12 (0.84)

Table 2: Transition analysis at the nitrogen K-edge. Excitation energies are given in eV. f is the oscillator strength and L is the lowest unoccupied molecular orbital (LUMO). Only transitions with the high amplitudes are given.

excitation center	excitation energy (f)	transition (amplitude)
$A-N7$	400.11 (0.042)	$1s \rightarrow$ L+0 (0.77)
$A-N3$	400.19 (0.046)	$1s \rightarrow$ L+1 (0.81)
$G-N7$	400.22 (0.044)	$1s \rightarrow$ L+3 (0.62)
$G-N3$	400.96 (0.033)	$1s \rightarrow$ L+3 (0.6)
$A-N6$	401.91 (0.02)	$1s \rightarrow$ L+0 (0.97)
$G-N2$	401.98 (0.018)	$1s \rightarrow$ L+6 (0.89)
$FU-N3$	402.19 (0.021)	$1s \rightarrow$ L+2 (0.94)
$A-N9$	402.34 (0.029)	$1s \rightarrow$ L+1 (0.77)
$A-N1$	402.58 (0.031)	$1s \rightarrow$ L+0 (0.86)
$G-N1$	402.72 (0.022)	$1s \rightarrow$ L+3 (0.79)
$G-N9$	402.86 (0.032)	$1s \rightarrow$ L+6 (0.86)
$FU-N1$	402.9 (0.019)	$1s \rightarrow$ L+2 (0.93)
$A-N6$	404.63 (0.015)	$1s \rightarrow$ L+8 (0.87)
$G-N1$	404.67 (0.027)	$1s \rightarrow$ L+14 (0.84)
$FU-N3$	405.36 (0.024)	$1s \rightarrow$ L+24 (0.46)
$A-N1$	405.43 (0.027)	$1s \rightarrow$ L+8 (0.81)
$A-N6$	405.47 (0.028)	$1s \rightarrow$ L+10 (0.6)
$FU-N1$	406.5 (0.018)	$1s \rightarrow$ L+15 (0.54)

Table 3: Transition analysis at the oxygen K-edge. Excitation energies are given in eV. f is the oscillator strength and L is the lowest unoccupied molecular orbital (LUMO). Only transitions with the high amplitudes are given.

excitation center	excitation energy (f)	transition (amplitude)
$FU-O4$	531.61 (0.038)	$1s \rightarrow$ L+2 (0.9)
$G-O6$	532.15 (0.027)	$1s \rightarrow$ L+3 (0.72)
$FU-O2$	532.91 (0.033)	$1s \rightarrow$ L+5 (0.95)
$O4''$	534.37 (0.018)	$1s \rightarrow$ L+11 (0.59)
$O2''$	534.7 (0.024)	$1s \rightarrow$ L+7 (0.86)
$A-O4'$	536.66 (0.028)	$1s \rightarrow$ L+13 (0.53)
$FU-O4'$	537.06 (0.028)	$1s \rightarrow$ L+16 (0.34)
$A-O5'$	537.24 (0.039)	$1s \rightarrow$ L+13 (0.7)
$FU-O3'$	537.25 (0.036)	$1s \rightarrow$ L+12 (0.47)
$O1''$	537.29 (0.043)	$1s \rightarrow$ L+17 (0.51)
$G-O5'$	537.45 (0.032)	$1s \rightarrow$ L+9 (0.34)
$FU-O4'$	537.68 (0.027)	$1s \rightarrow$ L+20 (0.24)

Table 4: Transition analysis at the fluorine K-edge. Excitation energies are given in eV. f is the oscillator strength and L is the lowest unoccupied molecular orbital (LUMO). Only transitions with the high amplitudes are given.

excitation center	excitation energy (f)	transition (amplitude)
$FU - F5$	688.9 (0.035)	$1s \rightarrow$ L+12 (0.57)

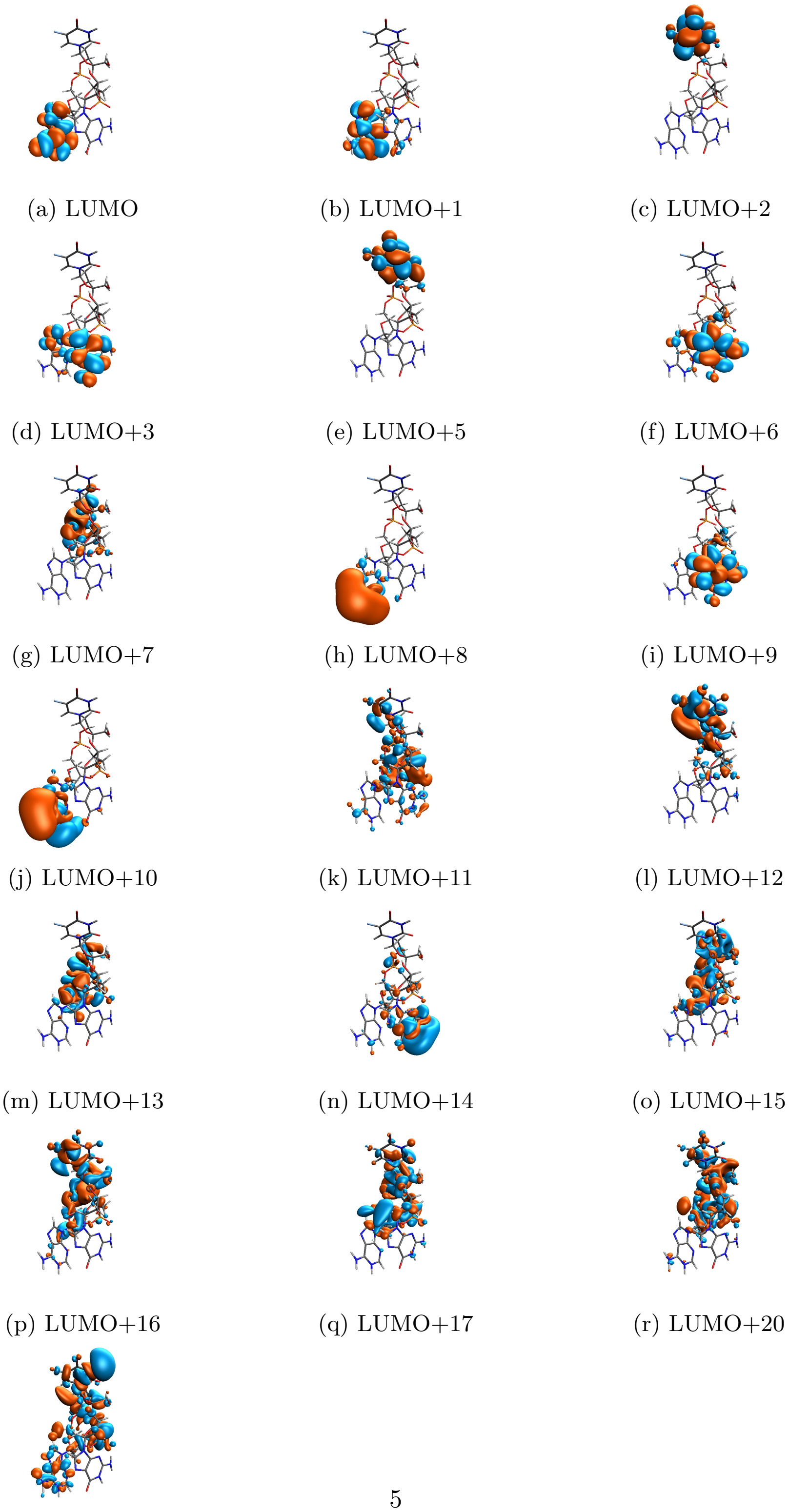

(a) LUMO (b) LUMO+1 (c) LUMO+2

(d) LUMO+3 (e) LUMO+5 (f) LUMO+6

(g) LUMO+7 (h) LUMO+8 (i) LUMO+9

(j) LUMO+10 (k) LUMO+11 (l) LUMO+12

(m) LUMO+13 (n) LUMO+14 (o) LUMO+15

(p) LUMO+16 (q) LUMO+17 (r) LUMO+20

(s) LUMO+24

Figure 1: Canonical orbitals for the most relevant excited states with isosurface of 0.05 au.
